# Food Insecurity and Hunger: Quiet Public Health Problems on Campus

**DOI:** 10.4172/2155-9600.1000668

**Published:** 2018

**Authors:** Michele R Forman, Lauren D Mangini, Yong-Quan Dong, Ladia M Hernandez, Karen L Fingerman

**Affiliations:** 1 Department of Nutrition Science, Purdue University, West Lafayette, IN 47907, USA; 2 Research Affiliate, Population Research Center, G1800, The University of Texas at Austin, Austin, TX 78712, USA; 3 Office of Medicine - Health Services Research, Baylor College of Medicine, Houston, TX 77030, USA; 4 Department of Nutritional Sciences, A2703, The University of Texas at Austin, Austin, TX 78712, USA; 5 Human Development and Family Sciences, A2702, The University of Texas at Austin, Austin, TX 78712, USA; 6 Population Research Center G1800, The University of Texas at Austin, Austin, TX 78712, USA

**Keywords:** Food insecurity, Postsecondary education, College campuses, Hunger, Financial skills

## Abstract

Food insecurity and hunger are gaining traction as recognized public health problems on college campuses in the United States. Data from recent publications and reports suggest the prevalence of food insecurity among U.S. undergraduate students ranges from 14.1 to 58.8%, compared to 12.3% of U.S. households. Undergraduate students (N=1,069) were surveyed at the University of Texas at Austin in 2014-2015. The survey questionnaire included the validated 6-item short-form of the USDA food security module, was distributed and completed in class and answered anonymously. The demographic characteristics of the sample are representative of all undergraduates on campus. Food insecurity was reported by 23.5% of students surveyed; ever being hungry by 31% and 12.5% of the ever-hungry also report being food-insecure. Importantly, most of the food insecure (96%) did not report experiencing food insecurity prior to matriculation. In multiple logistic regression models, the factors associated with a higher odds ratio of food insecurity include: being a first-generation college student; Hispanic ethnicity; third-born child or later in the family; and less confident about financial management skills. The factors associated with a higher adjusted odds ratio of hunger include: being of Asian or other ethnicity (vs. non-Hispanic white) and having limited confidence about financial management skills. The results, from one of the largest surveys of food insecurity and hunger among undergraduate students on a single campus in the U.S., suggest that transition to college is a vulnerable window for the emergence of food insecurity and hunger. More research is needed on the long-term effects of food insecurity in this population and the effectiveness of campus policy and interventions addressing food insecurity and hunger.

## Introduction

Food insecurity exists when there is “a limited or uncertain availability of nutritionally adequate or safe foods or limited or uncertain ability to acquire food in socially acceptable ways” [1]. In the United States, 12.3% of households were food-insecure in 2016 [2]. Food insecurity is associated with physical and psychological health problems at multiple stages of the life course, including: lower parent-rated physical health and more hospitalizations in infants and toddlers; impaired academic and social skills and asthma in young children; anemia and lower bone-mineral density in adolescents under 18 y; and chronic conditions such as obesity, hypertension, diabetes and the metabolic syndrome in adults [3-14]. Food insecurity disproportionately affects ethnic minorities, low-income households, people of low educational status, and those lacking health insurance, mirroring the general pattern of health disparities observed in America [15-17]. Although rates of food insecurity are higher in low-income populations (those of lower socioeconomic status), detrimental health outcomes associated with food insecurity persist even when socioeconomic status is accounted for, demonstrating that food insecurity is a unique, pressing threat to public health in the United States [18-20].

Despite the increase in studies on food insecurity in the last decade, research on university undergraduates lags behind that focused on other populations; investigators typically collect and utilize data on children under 18 y or adults living in familial households. A 2016 survey of nearly 9,000 University of California (UC) students across 10 campuses found that 39% had low or very low food security [21]. Another publication, the most comprehensive review of peer-reviewed and “gray” (published but not peer-reviewed) literature to date, summarized findings from 59 works, including 9 that were peer-reviewed and U.S.-based. The latter studies yielded an average prevalence of food insecurity of 32.9% in U.S. undergraduates, with a range of 14.1 to 58.8% [22].

These findings send a red flag: the reported prevalence of food insecurity in these populations of young adult students is alarmingly higher than nationally reported data for U.S. households. These studies and reports are the first published on this population, which dwells in the lacuna of food insecurity research. Although food security status has been investigated as a factor in children’s school performance, interest has largely been limited to students in elementary or middle school [6,23]. However, the UC report states that food-insecure students self-reported a lower grade-point average (GPA) and more financial hardship than food-secure students, indicating that food insecurity likely has consequences for school performance in this age group and should be investigated in more depth, using validated tools [21].

Hunger is frequently mistaken for food insecurity but relates to a biological state of food insufficiency leading to a lack of satiety and need for more food sources [20]. Hunger is typically recognized under conditions like states of famine, immigration and political unrest and when resources are at a bare minimum; it is usually considered a global issue targeted to economically disadvantaged populations, not university campuses in the U.S. Yet, a recent Canadian report estimates a prevalence of 25% hunger among school attendees aged 11-15 y. The prevalence estimate was based on response to a single question whether the student went to school or to bed hungry due to a lack of food in the home at least occasionally or often [24]. Canadian studies document associations between hunger and depression, as well as other chronic conditions such as asthma, in adolescents [25]. With these data from a neighboring developed country, the need to assess hunger among college students in the U.S. becomes evident.

The objectives of this research were to: estimate the prevalence of food insecurity and hunger among undergraduate students; and identify the factors associated with food insecurity and with hunger in the student population attending the University of Texas at Austin.

## Materials and Methods

The study was designed as a cross-sectional survey of undergraduate students at one point during the school year with data collection starting the fall semester of 2014 through the fall semester of 2015. The sample was drawn from students enrolled in the Colleges of Liberal Arts and of Natural Sciences, two of the largest colleges at the University of Texas at Austin (UT). The primary means of recruitment was email announcements sent by the Principal Investigator (PI) (MRF) to department chairs in the colleges. The email identified the high prevalence of food insecurity among college students in a pilot survey at UT during the prior year and in earlier surveys and indicated the concern for potential adverse effects from food insecurity and hunger on academic performance and physical and mental health among undergraduate students at UT [26,27]. If the department chairs agreed to help, then the chairs identified the largest class(es) (size and faculty responsible for teaching sections with dates and times for class/sections). The research assistant (LDM) from the PI’s laboratory who brought the 10-15 minute survey questionnaires to the sections explained the intent of the survey, that the responses would be anonymous and that participation was voluntary. The total sample of participating students was 1069, representing 99.5% completion. The primary data collection tool of this study was the validated 6-Item Short-Form of the Household Food Security Survey Module [28]. These 6 questions address an individual’s food situation, with positive responses to two items required for classification as food insecure. In addition, two items that ask about previous (pre-matriculation) experience with food insecurity were drawn directly from a validated screener for food insecurity [29]. The questionnaire also assessed: sociodemographic characteristics of the individual (age, sex, race, birth order, employment, financial aid receipt); confidence in financial management skills, which has been associated with outcomes related to food insecurity; and the individual’s experience with hunger at UT [30]. The answers to these questions were intended to distinguish the prevalence of food insecurity (a psychological phenomenon with detrimental nutritional and health outcomes) from that of hunger (a distinct physiological occurrence) [1]. Furthermore, the questionnaire included a set of items on family (financial) support and its direction (parent/guardian to child; child to parent/guardian). These items have been published and were intended to examine any relationship between food insecurity and/or hunger and the degree of family support provided to the adolescent [31,32]. Study data *i.e.* questionnaire responses were collected, doubly entered with visual reviews to assess data entry errors using REDCap electronic data capture tools at UT Austin [33]. All survey forms are kept in a locked secure location. The Institutional Review Board at the University of Texas approved this study. Students implied consent to the questionnaire by reading and completing it.

### Statistical analysis

We estimated the prevalence of food security and of hunger as well as sociodemographic characteristics of the entire sample. We conducted bivariate analyses using Pearson’s χ^2^ statistic to assess the associations between food security/insecurity and hunger by sociodemographic variables. We generated crude and adjusted odds ratios (OR) with 95% confidence intervals (95% CI) for food insecurity and for hunger using multiple logistic regression models adjusting for the sociodemographic covariates. The level of significance for all statistical tests was set at a *P* value of <0.05.

## Results

Among 1069 students, 58% are women, 17% first generation university attendees, and are represented by the following percentages of each race-ethnicity group: 42% non-Hispanic White (NHW), 3% non-Hispanic Black (NHB), 21% Hispanic, 23% Asian and 9% “other” race-ethnicity (Table 1). Among the participants, 47% received university financial aid, 23% were currently working, and 50% reported paying for part or all their living expenses (*e.g.* housing, food). About eight percent were only-children, 41% were the eldest sibling in the family, while 32% were second-born and 16% were third or later births. The mean/median age of the participants was 19 ± 2 y.

Approximately 23.5% of students responded positively to 2 items, indicating food insecurity (FIS), with an equal proportion (23.8%) affirmative among males and females. Among the race-ethnicity groups, over 30 percent of NHB and Hispanic students reported FIS, with a smaller percentage of the Asian (23%) and the lowest percentage of the NHW (19%) affirmative for FIS. Only 4% of the food-insecure reported being FIS prior to university.

Since attending university, 26.6% ever cut the size of the meal or skipped meals because money was insufficient for food. Importantly, 37% of these respondents reported doing so almost every month. Fifteen percent reported ever being hungry and did not eat because the student could not afford food. In another item, 31% reported ever hungry because they did not have enough food, and 12.5% of these students were both FIS and hungry. The 31% who reported ever-hungry with insufficient food were the focus of subsequent analysis of factors associated with hunger.

A higher proportion of students who reported FIS were: third-born or higher; first generation college student; worked for pay; were Hispanic; were not at all or were only a little confident about his/her ability to manage finances; did not have parents who paid tuition nor paid for living expenses and therefore did so; and had financial aid at university (Table 2).

The demographic characteristics and financial factors related to the adjusted odds ratio of FIS appear in Table 3A. Compared to only children, a student who was the third born or later had over a two-fold increased odds of FIS. Compared to those who are not first-generation students, those who were first generation college students were at a 59% higher odds of FIS.

Compared to NHWs, Hispanic students had a 90% higher odd of FIS. Compared to students who were confident in their ability to manage finances, those who were not confident or who had little confidence had over a two-fold higher odds of FIS (OR=2.51, 95% CI 1.05-6.05; OR=2.43 95% CI 1.33-4.41, respectively). The remaining covariates including working for pay and parental support for tuition or for living expenses were not associated with the odds of FIS in the multivariate models.

The demographic characteristics and financial factors related to the adjusted odds of hunger appear in Table 3B. Compared to NHWs, Asian and students in the “other” race-ethnicity groups had a 50% to 2-fold higher odds of going hungry. Compared to students who were confident in their ability to manage finances, those who were somewhat or a little confident in their ability to manage finances had a two-fold higher odds of being hungry (OR=2.10 95% CI 1.20-3.69; OR=2.15 95% CI 1.34-3.45, respectively). Compared to those did not have to pay for their living expenses, those who did have to pay for expenses were at a 66% higher odds of being hungry.

## Discussion

Among undergraduate students at the University of Texas at Austin, 23.5% reported FIS, with a disproportionately higher percentage of FIS among the Hispanic than NHW or Asian students. Importantly only four percent of the FIS reported experiencing FIS prior to attending the university: that is a clear flag for the transition to college as a vulnerable period for FIS among young adults. The factors associated with the higher adjusted odds of FIS included: being a first-generation college student, Hispanic ethnicity, a third born child or later in the family, and less confident about financial management. Approximately 31% of the students reported being hungry and 12.5% of these students reported being both hungry and FIS. The factors associated with the higher adjusted odds of hunger included: being of Asian or “other” race-ethnicity and limited confidence about financial management.

The prevalence of FIS at UT (23.5%) falls within the range among university students in earlier surveys [21,22]. The disproportionate representation of FIS among Hispanic and NHB undergraduate students mirrors the race-ethnic distribution of FIS in national data and also appeared in a 2015 report on FIS at UC campuses [21,34]. To the best of our knowledge, no other publication has reported the prevalence of hunger in university students and of both hunger and FIS. The presence of both hunger and FIS heightens the concern for adverse ripple effects impacting academic performance, likelihood of graduation, and mental and physical health. Research on FIS and adverse health conditions including obesity, asthma, and sleep disturbance is well-documented for children and adults but not in this demographic sub-population [8,35-38]. There was a low percentage who reported FIS prior to college, similar to the UC findings that the majority of the FIS were food-secure prior to matriculation [21]. These results herald the potential for transition to college as both a period of vulnerability for FIS and an opportunity for development of prevention strategies early in adulthood.

Among the factors associated with FIS, being a first-generation college student and a birth order of three or later has not been previously reported. The constellation of demographic factors including race-ethnicity could be used to screen incoming students for high risk of FIS while the inability to manage finances might trigger a plan to develop management skills prior to or during freshman orientation. Few factors were associated with hunger, but once again an inability to manage finances arose as a risk factor that could be ameliorated through early development of skills. Prior evidence suggests targeting financial management skills may impact risk of food insecurity [30,39,40]. More research is warranted to identify the complex set of social, economic and developmental origins behind FIS and hunger at this stage of the life course. Much of the challenge in addressing these phenomena on campus will likely arise from tracking health and academic consequences without stigmatizing students.

The survey has strengths and limitations. While the sample was based on the willingness for department chairs and their faculty to agree for the survey administration in their large classes and volunteer student participation, the sample was representative of UT students in 2016 by race-ethnicity (41% NHW, 21% Hispanic, 5% NHB, 23% Asian) and sex (54% female) [41]. The large sample size of over 1,000 students compared with some prior surveys was a strength, as was the use of a validated short-form of the USDA 18-item food security survey. Because the survey was administered during class, high completion rates were attained with fewer than five students not completing the questionnaire. Yet the brevity of the survey questionnaire limited the range of data from students, thus, information about sleep habits, physical activity, and other lifestyle behaviors as well as health comorbidities were not collected. Finally, the study was a cross-sectional survey without followup of the FIS/hungry and probable associations with academic, or other dimensions of, performance such as GPA [21].

In summary, FIS and hunger are significant problems among undergraduate students attending a large public university in the southwestern U.S. Recognizing their vulnerability as they transition to the adult years provides an opportunity for preventive approaches and intervention during college to address FIS and health disparities in the U.S. Food availability should be expanded directly, *e.g.* via food pantries or campus gardens, and indirectly through reduced student fees. The latter represents less visible strategies that may ameliorate conditions while maintaining confidentiality around FIS and hunger. Of heightened importance should be the focus of food access to nutritious foods to reduce the potential for obesity and other adverse health conditions. Student health services might explore the means to screen for FIS, similar to the approach taken by the American Academy of Pediatrics recommendation for pediatricians to screen for FIS during visits [42]. Further research is needed to address the consequences of FIS and hunger during transition to adult years and estimate the prevalence of both conditions on other campuses across the U.S.

## Figures and Tables

**Table 1:** Demographic characteristics of 1069 student respondents at UT Austin 2014-15.

Variable	% (N)
Sex	
Male	39 (420)
Female	58 (616)
First generation university student	
Yes	17 (179)
No	83 (884)
Race-ethnicity	
Non-Hispanic White	42 (444)
Non-Hispanic Black	3 (35)
Hispanic	21 (220)
Asian	23 (251)
Other	9 (98)
Receive financial aid at university	
Yes	47 (505)
No	52 (556)
Currently working	
Yes	23 (241)
No	77 (824)
Birth order	
Only	8 (85)
First-born	41 (442)
Second-born	32 (337)
Third or higher born	16 (169)

**Table 2:** The percentage (N) of food security and insecurity by demographic characteristics.

	Food secure (N=818)	Food insecure (N=251)	P(χ^2^)
**Birth order**			
Firstborn	44. 0 (349)	38.8 (93)	
Second-born	32.4 (257)	33.3 (80)	
Third-born or higher	14.4 (114)	22.9 (55)	
Only child	9.2 (73)	5.0 (12)	<0.01
**Sex**			
Male	40.6 (320)	40.5 (100)	
Female	59.4 (469)	59.5 (147)	0.98
**First-generation college/university student**			
Yes	13.9 (113)	26.4 (66)	
No	86.1 (700)	73.6 (184)	<0.01
**Working for pay**			
Yes	21.1 (173)	27.3 (68)	
No	78.8 (643)	72.7 (181)	0.04
**Race/ethnic group**			
Non-Hispanic White	44.6 (358)	35.1 (86)	
Non-Hispanic Black	3.0 (24)	4.5 (11)	
Hispanic, any race	18.1 (145)	30.6 (75)	
Asian	24.4 (196)	22.4 (55)	
Other	9.9 (80)	7.4 (18)	<0.01
**How confident are you in your ability to manage your finances/money?**			
Not confident	2.7 (22)	5.2 (13)	
A little confident	10.5 (86)	16.8 (42)	
Somewhat confident	31.4 (256)	34.8 (87)	
Confident	40.1 (327)	32.8 (82)	
Very confident	15.3 (125)	10.4 (26)	<0.01
**Parents currently help pay tuition or fees for you to attend college?**			
Yes	81.0 (660)	69.9 (174)	
No	19.0 (155)	30.1 (75)	<0.01
**Parents contribute funds for your living expenses (e.g. paying for a dorm or apartment, money for food)?**			
Yes	87.1 (710)	80.3 (200)	
No	12.9 (105)	19.7 (49)	<0.01
**Do you pay for part (or all) of your own tuition or fees to attend college?**			
Yes	35.0 (285)	45.2 (112)	
No	65.0 (529)	54.8 (136)	<0.01
**Do you pay for part (or all) of your living expenses (e.g. paying for a dorm or apartment, money for food)?**			
Yes	48.0 (391)	60.2 (150)	
No	52.0 (423)	39.8 (99)	<0.01
**Financial aid at UT**			
Yes	45.4 (369)	54.6 (136)	
No	54.6 (443)	45.4 (113)	0.01

**Table 3A:** Adjusted odds ratio (OR) and 95% CI for food insecurity by demographic characteristics.

	OR (95% CI)	P
**Birth order**		
Firstborn	1.61 (0.80-3.23)	
Second-born	1.87 (0.93-3.77)	
Third-born or higher	2.78 (1.33-5.84)	
Only child	Reference	0.02
**First-generation college/university student**		
Yes	1.59 (1.05-2.41)	
No	Reference	0.03
**Working for pay**		
Yes	Reference	
No	0.83 (0.57-1.21)	0.34
**Race/ethnic group**		
Non-Hispanic White	Reference	
Non-Hispanic Black	1.84 (0.79-4.27)	
Hispanic, any race	1.90 (1.26-2.87)	
Asian	1.26 (0.83-1.91)	
Other	0.87 (0.47-1.61)	0.02
**How confident are you in your ability to manage your finances/money?**		
Not confident	2.51 (1.05-6.05)	
A little confident	2.43 (1.33-4.41)	
Somewhat confident	1.52 (0.91-2.53)	
Confident	1.04 (0.63-1.74)	
Very confident	Reference	<0.01
**Parents currently help pay tuition or fees for you to attend college?**		
Yes	Reference	
No	1.14 (0.75-1.74)	0.54
**Do you pay for part (or all) of your own tuition or fees to attend college?**		
Yes	Reference	
No	0.77 (0.52-1.12)	0.18
**Do you pay for part (or all) of your living expenses (e.g. paying for a dorm or apartment, money for food)?**		
Yes	Reference	
No	0.82 (0.56-1.19)	0.29
**Financial aid at UT**		
Yes	Reference	
No	1.13 (0.80-1.61)	0.49

**Table 3B:** Adjusted odds ratio (OR) and 95% CI for hunger by demographic characteristics.

	OR (95% CI)	P
**Birth order**		
Firstborn	1.20 (0.70-2.06)	
Second-born	1.29 (0.74-2.23)	
Third-born or higher	0.81 (0.44-1.51)	
Only child	Reference	0.19
**First-generation college/university student**		
Yes	Reference	
No	1.04 (0.70-1.55)	0.83
**Working for pay**		
Yes	Reference	
No	1.07 (0.76-1.52)	0.70
**Race/ethnic group**		
Non-Hispanic White or Caucasian	Reference	
Non-Hispanic Black	0.91 (0.39-2.15)	
Hispanic, any race	1.23 (0.83-1.81)	
Asian	1.53 (1.06-2.19)	
Other	2.37 (1.47-3.84)	<0.01
**How confident are you in your ability to manage your finances/money?**		
Not confident	1.94 (0.83-4.54)	
A little confident	2.10 (1.20-3.69)	
Somewhat confident	2.15 (1.34-3.45)	
Confident	1.50 (0.94-2.39)	
Very confident	Reference	0.01
**Parents currently help pay tuition or fees for you to attend college?**		
Yes	Reference	
No	1.46 (0.99-2.15)	0.06
**Do you pay for part (or all) of your own tuition or fees to attend college?**		
Yes	Reference	
No	1.26 (0.88-1.79)	0.21
**Do you pay for part (or all) of your living expenses (e.g. paying for a dorm or apartment, money for food)?**		
Yes	1.66 (1.18-2.32)	
No	Reference	<0.01
**Financial aid at UT**		
Yes	Reference	
No	0.91 (0.66-1.24)	0.53

## Abbreviations:

CI: Confidence Interval
FIS: Food Insecurity, Food-Insecure
GPA: Grade-Point Average
NHB: Non-Hispanic Black
NHW: Non-Hispanic White
PI: Principal Investigator
OR: Odds Ratio
UC: University of California
UT: University of Texas

## References

[1] https://www.ers.usda.gov/topics/food-nutrition-assistance/food-security-in-the-us/measurement.aspx#.Udr58FNQ36Y

[2] Coleman-Jensen A, Rabbitt MP, Gregory CA, Singh A (2017) Household Food Security in the United States in 2016: United States Department of Agriculture, p: 44.

[3] Laraia BA, Siega-Riz AM, Gundersen C (2010) Household food insecurity is associated with self-reported pregravid weight status, gestational weight gain, and pregnancy complications. J Am Diet Assoc 110: 692–701. 20430130 10.1016/j.jada.2010.02.014 PMC3018748

[4] Cook JT, Frank DA, Berkowitz C, Black MM, Casey PH, (2004) Food insecurity is associated with adverse health outcomes among human infants and toddlers. J Nutr 134: 1432–1438. 15173408 10.1093/jn/134.6.##

[5] Casey PH, Szeto KL, Robbins JM, Stuff JE, Connell C, (2005) Child health-related quality of life and household food security. Arch Pediat Adol Med 159: 51–56. 10.1001/archpedi.159.1.51 15630058

[6] Jyoti DF, Frongillo EA, Jones SJ (2005) Food insecurity affects school children’s academic performance, weight gain, and social skills. J Nutr 135: 2831–2839. 16317128 10.1093/jn/135.12.2831

[7] Howard LL (2011) Transitions between food insecurity and food security predict children’s social skill development during elementary school. Br J Nutr 105: 1852–1860. 21269533 10.1017/S0007114510005623

[8] Mangini LD, Hayward MD, Dong YQ, Forman MR (2015) Household food insecurity is associated with childhood asthma. J Nutr 145: 2756–2764. 26491120 10.3945/jn.115.215939

[9] Eicher-Miller HA, Mason AC, Weaver CM, McCabe GP, Boushey CJ (2009) Food insecurity is associated with iron deficiency anemia in US adolescents. Am J Clin Nutr 90: 1358–1371. 19776137 10.3945/ajcn.2009.27886

[10] Eicher-Miller HA, Mason AC, Weaver CM, McCabe GP, Boushey CJ (2011) Food insecurity is associated with diet and bone mass disparities in early adolescent males but not females in the United States. J Nutr 141: 1738–1745. 21795427 10.3945/jn.111.142059

[11] Stuff JE, Casey PH, Szeto KL, Gossett JM, Robbins JM, (2004) Household food insecurity is associated with adult health status. J Nutr 134: 2330–2335. 15333724 10.1093/jn/134.9.2330

[12] Parker ED, Widome R, Nettleton JA, Pereira MA (2010) Food security and metabolic syndrome in U.S. adults and adolescents: findings from the National Health and Nutrition Examination Survey, 1999-2006. Ann Epidemiol 20: 364–370. 20382337 10.1016/j.annepidem.2010.02.009 PMC5842796

[13] Pan L, Sherry B, Njai R, Blanck HM (2012) Food insecurity is associated with obesity among US adults in 12 states. J Acad Nutr Diet 112: 1403–1409. 22939441 10.1016/j.jand.2012.06.011 PMC4584410

[14] Seligman HK, Laraia BA, Kushel MB (2010) Food insecurity is associated with chronic disease among low-income NHANES participants. J Nutr 140: 304–310. 20032485 10.3945/jn.109.112573 PMC2806885

[15] Coleman-Jensen A, Rabbitt MP, Gregory CA, Singh A (2016) Household food security in the United States in 2015. Washington, DC: United States Department of Agriculture; Economic Research Service, p: 44.

[16] Schuster MA, Elliott MN, Kanouse DE, Wallander JL, Tortolero SR, (2012) Racial and ethnic health disparities among fifth-graders in three cities. N Engl J Med 367: 735–745. 22913683 10.1056/NEJMsa1114353

[17] Lantz PM, House JS, Mero RP, Williams DR (2005) Stress, life events, and socioeconomic disparities in health: results from the Americans’ changing lives study. J Health Soc Behav 46: 274–288. 16259149 10.1177/002214650504600305

[18] Gundersen C (2013) Food insecurity is an ongoing national concern. Adv Nutr 4: 36–41. 23319121 10.3945/an.112.003244 PMC3648737

[19] Gundersen C, Kreider B, Pepper J (2011) The economics of food insecurity in the United States. Appl Econ Perspect Policy 33: 281–303.

[20] Alaimo K (2005) Food insecurity in the United States: an overview. Top Clin Nutr 20: 281–298.

[21] Martinez SM, Maynard K, Ritchie LD (2016) Student food access and security survey. Oakland, CA: University of California, p: 29.

[22] Bruening M, Argo K, Payne-Sturges D, Laska MN (2017) The struggle is real: a systematic review of food insecurity on postsecondary education campuses. J Acad Nutr Diet 117: 1767–1791. 28754200 10.1016/j.jand.2017.05.022 PMC6901286

[23] Burke MP, Jones SJ, Fram MS, Frongillo EA (2012) U.S. households with children are exposed to nonpersistent and persistent food insecurity. J Hunger Environ Nutr 7: 349–362.

[24] Pickett W, Michaelson V, Davison C (2015) Beyond nutrition: hunger and its impact on the health of young Canadians. Int J Public Health 60: 527–538. 25929577 10.1007/s00038-015-0673-z PMC4480846

[25] Ke J, Ford-Jones EL (2015) Food insecurity and hunger: A review of the effects on children’s health and behaviour. Paediatr Child Health 20: 89–91. 25838782 10.1093/pch/20.2.89 PMC4373582

[26] Patton-Lopez MM, Lopez-Cevallos DF, Cancel-Tirado DI, Vazquez L (2014) Prevalence and correlates of food insecurity among students attending a midsize rural university in Oregon. J Nutr Educ Behav 46: 209–214. 24406268 10.1016/j.jneb.2013.10.007

[27] Chaparro MP, Zaghloul SS, Holck P, Dobbs J (2009) Food insecurity prevalence among college students at the University of Hawai’i at Manoa. Public Health Nutr 12:2097–2103. 19650961 10.1017/S1368980009990735

[28] Blumberg SJ, Bialostosky K, Hamilton WL, Briefel RR (1999) The effectiveness of a short form of the household food security scale. Am J Public Health 89: 1231–1234. 10432912 10.2105/ajph.89.8.1231 PMC1508674

[29] Hager ER, Quigg AM, Black MM, Coleman SM, Heeren T, (2010) Development and validity of a 2-item screen to identify families at risk for food insecurity. Pediatrics 126: e26–32. 20595453 10.1542/peds.2009-3146

[30] Gundersen CG, Garasky SB (2012) Financial management skills are associated with food insecurity in a sample of households with children in the United States. J Nutr 142: 1865–1870. 22955515 10.3945/jn.112.162214

[31] Fingerman KL, Cheng YP, Kim K, Fung H, Hae G, Parental support of college students in Germany, Hong Kong, Korea, and the US. J Fam Issues 2016;37: 1384–1411. 27594722 10.1177/0192513X14541444 PMC5007000

[32] Sweet J, Bumpass L, Call V (1988) The design and content of the national survey of families and households. Madison, WI: University of Wisconsin-Madison.

[33] Harris PA, Taylor R, Thielke R, Payne J, Gonzalez N, (2009) Research electronic data capture (REDCap)--a metadata-driven methodology and workflow process for providing translational research informatics support. J Biomed Inform 42: 377–381. 18929686 10.1016/j.jbi.2008.08.010 PMC2700030

[34] https://www.ers.usda.gov/topics/food-nutrition-assistance/food-security-in-the-us/key-statistics-graphics.aspx#.Uebsr1O9xSU.

[35] Ding M, Keiley MK, Garza KB, Duffy PA, Zizza CA (2015) Food insecurity is associated with poor sleep outcomes among US adults. J Nutr 145: 615–621. 25733479 10.3945/jn.114.199919

[36] Dinour LM, Bergen D, Yeh MC (2007) The food insecurity-obesity paradox: a review of the literature and the role food stamps may play. J Am Diet Assoc 107: 1952–1961. 17964316 10.1016/j.jada.2007.08.006

[37] Gooding HC, Walls CE, Richmond TK (2011) Food insecurity and increased BMI in young adult women. Obesity 20: 1896–1901. 21779092 10.1038/oby.2011.233 PMC4248561

[38] Leung CW, Williams DR, Villamor E (2012) Very low food security predicts obesity predominantly in California Hispanic men and women. Public Health Nutr 15:2228–2236. 22463949 10.1017/S1368980012000857 PMC3502688

[39] Olson CM, Anderson K, Kiss E, Lawrence FC, Selling SB (2004) Factors protecting against and contributing to food insecurity among rural families. Fam Econ Nutr Rev 16: 12–20.

[40] Gaines A, Robb CA, Knol LL, Sickler S (2014) Examining the role of financial factors, resources and skills in predicting food security status among college students. Int J Consum Stud 38: 374–384.

[41] https://admissions.utexas.edu/explore/freshman-profile.

[42] Pediatrics CoC, Nutrition Co (2015) Promoting food security for all children. Pediatrics 136: e1431–1438. 26498462 10.1542/peds.2015-3301

